# Universal Screening for Familial Hypercholesterolemia in Preschool Children and Their Families in Slovenia (FH-FAMILIES)—A Protocol for a Study of Four-Stage Screening Program

**DOI:** 10.3390/jpm15110510

**Published:** 2025-10-29

**Authors:** Mia Becker, Bernarda Vogrin, Jan Kafol, Barbara Čugalj Kern, FH-FAMILIES Collaborators, Urh Grošelj

**Affiliations:** 1 Faculty of Medicine, University of Maribor, 2000 Maribor, Slovenia; mia.becker@student.um.si (M.B.); bernarda.vogrin@hotmail.com (B.V.); 2 Public Health Center Maribor, 2000 Maribor, Slovenia; 3 Outpatient Clinic Lenart, 2230 Lenart, Slovenia; 4 Department f Vascular Diseases, UMC Ljubljana, 1000 Ljubljana, Slovenia; 5 Clinical Institute for Special Laboratory Diagnostics, University Children’s Hospital, UMC Ljubljana, 1000 Ljubljana, Slovenia; barbara.kern@kclj.si; 6 Department of Pediatric Endocrinology, Diabetes and Metabolic Disease, University Children’s Hospital, UMC Ljubljana, 1000 Ljubljana, Slovenia; 7 Faculty of Medicine, University of Ljubljana, 1000 Ljubljana, Slovenia

**Keywords:** hypercholesterolemia, universal screening, preschoolers, total cholesterol, genetic testing

## Abstract

Familial hypercholesterolemia (FH) is the most common metabolic disease, with prevalence estimated between 1:250 and 1:300. The affected individuals have a significantly higher risk for developing atherosclerosis and cardiovascular disease (CVD) compared to non-affected individuals. Early CVD can be prevented with early detection and treatment of FH. In Slovenia we have been conducting a national three-staged program of universal screening for FH of preschoolers. **Goals:** Our goal is to collect data for 5000 children, which is approximately one-quarter of one generation of preschoolers for the year 2023 (*n* = 5000). **Methods**: Our study includes both prospective and retrospective components and is a non-interventional cohort study. The prospective component began in 2023, when a questionnaire was distributed to multiple community health centers and outpatient practices in Slovenia. Pediatricians or school medicine specialists completed these questionnaires. The retrospective component involves our research team collecting the remaining necessary data from existing medical records. We are going to follow our algorithm for the implementation of the universal cholesterol screening program and seek all children that will be referred to the Pediatric Lipid Clinic at the University Children’s Hospital, University Medical Centre (UCH-UMC), Ljubljana, for further genetic testing. If a child has a positive genetic result, their parents and siblings will undergo genetic testing. **Conclusions:** Despite being a common genetic disorder, familial hypercholesterolemia (FH) is still largely underdiagnosed globally; fewer than 10% of affected individuals are thought to be identified. Early detection through effective screening is therefore essential to improve outcomes and prevent premature cardiovascular events.

## 1. Introduction

Atherosclerotic cardiovascular disease (ASCVD) and clinical manifestations, such as myocardial infarction and ischemic stroke, are the leading causes of morbidity and mortality worldwide [1]. In industrialized countries, one-quarter of all adult deaths result from arterial blockages caused by atherosclerotic plaques. More specifically, elevated low-density lipoprotein (LDL) concentrations have been the third most significant risk factor for developing cardiovascular disease (CVD) [2].

Primary hypercholesterolemia refers to elevated plasma concentrations of LDL resulting from genetic defects in lipoprotein metabolism. The condition encompasses both monogenic and polygenic forms [3]. The most common monogenic cause is FH, which is typically inherited in an autosomal dominant manner and results from pathogenic variants in the *LDLR*, *APOB*, or *PCSK9* genes, leading to impaired LDL receptor–mediated clearance of plasma LDL particles. Homozygous FH is rare and presents with markedly elevated LDL-C levels and early-onset atherosclerotic cardiovascular disease, often in childhood or adolescence [4].

In contrast, polygenic hypercholesterolemia arises from the cumulative effect of multiple common alleles that each modestly increase LDL levels. Although less severe than monogenic FH, the polygenic form is much more prevalent in the general population and still contributes significantly to the global burden of cardiovascular disease [5].

In addition, children worldwide are increasingly adopting unhealthy lifestyles, characterized by more sedentary behavior, consumption of calorie-dense foods with low nutritional value, and sugary drinks. These factors add to other risks for developing hypercholesterolemia, atherosclerosis, and CVD. Therefore, early screening of children for dyslipidemia and FH is becoming increasingly important as a primary preventive measure against atherosclerosis and CVD [6].

Effective lowering of LDL cholesterol can substantially reduce the risk of cardiovascular disease in individuals with familial hypercholesterolemia. The earlier the condition is identified, and treatment is initiated, and the more consistently therapy is maintained, the greater the clinical benefit. Consequently, childhood represents the optimal period for the detection and management of FH [7].

Hypercholesterolemia may manifest as a feature of other cholesterol-metabolism disorders. These include, for example, Wolman disease, sitosterolemia, and cholesteryl ester storage disease, as well as various inherited forms of hypertriglyceridemia, hypobetalipoproteinemia, hyperchylomicronemia, and hypoalphalipoproteinemia. These phenotypes are associated with pathogenic variants in genes such as *ABCA1*, *ABCG5*, *ALMS1*, *APOA1*, *APOA5*, *APOC2*, *APOC3*, *APOE*, *CREB3L3*, *GPIHBP1*, *LDLRAP1*, *LIPA*, *LMF1*, and *LPL*, and are classified among the rare dyslipidemias frequently encountered in the monogenic dyslipidemia literature (e.g., EAS consensus) [8,9].

Secondary hypercholesterolemia arises from various acquired conditions including endocrine disorders (such as hypothyroidism, Cushing’s syndrome, diabetes), renal disease (e.g., nephrotic syndrome, chronic kidney disease), hepatobiliary disease (e.g., cholestasis), certain pharmacologic agents (e.g., glucocorticoids, cyclosporine, some diuretics), and lifestyle or nutritional factors. Recent consensus statements emphasize that hypothyroidism is one of the most frequent endocrine causes, with increased LDL, delayed LDL clearance, and sometimes mild elevations in triglycerides, which may partially normalize with restoration of thyroid function [10,11,12].

Both monogenic and polygenic forms of primary hypercholesterolemia are collectively responsible for a large proportion of cases of premature ASCVD. Early identification through lipid screening and genetic testing, followed by appropriate lifestyle modification and pharmacologic therapy, is therefore essential to mitigate long-term cardiovascular risk [4].

### 1.1. Prevalence

Health impact is evaluated using the disability-adjusted life year (DALY), an indicator reflecting the total disease burden in terms of years lost to ill health, disability, or premature death. In 2019, elevated LDL was estimated to account for 4.40 million (95% CI: 3.30–5.65 million) deaths [1]. The prevalence of heterozygous FH is estimated to be between 1:250 and 1:300; in countries with high rates of consanguinity, even higher [13]. A worldwide meta-analysis of 11 million subjects published a prevalence of 1:313 [14]. Homozygous FH (HoFH) is a rare disease with an estimated prevalence of 1: 1,000,000 [15].

### 1.2. Genetics

FH is an autosomal dominant genetic disorder characterized by elevated concentrations of total cholesterol (TC) and/or LDL cholesterol. This results from either a reduced number of low-density lipoprotein receptor (LDLR) molecules or decreased LDLR activity, leading to impaired clearance of LDL-C from the plasma and consequently increased plasma concentrations of LDL-C and TC [16]. FH is primarily caused by pathogenic variants in the gene encoding the low-density lipoprotein receptor—*LDLR*. Less commonly, pathogenic variants occur in the apolipoprotein B gene (*APOB*) and proprotein convertase subtilisin/kexin type 9 genes (*PCSK9*), while in very rare cases, homozygous recessive variantsin the *LDLRAP1* gene are responsible [17]. The LDLR on the cell surface, upon recognizing ABOB—the main protein component of LDL—induces endocytosis of the LDL–LDLR complex. Cholesterol from the LDL particles is then released inside the cells, while the LDLR can be recycled back to the cell surface. FH can also be caused by variants in the receptor-binding domain of *APOB*, which reduce the ability of LDL particles to bind to LDLR, as well as by a rarer gain-of-function variants in the *PCSK9* gene. Functional PCSK9 prevents the recycling of LDLR back to the surface of hepatocytes, thereby reducing its concentration on the cell surface and consequently decreasing the uptake of atherogenic LDL particles [18].

Most individuals with HoFH have extreme hypercholesterolemia with accelerated atherosclerosis, if left untreated. The clinical picture depends on the proportion of preserved LDLR activity [15,19].

### 1.3. Diagnosis

Diagnosis can be established using various diagnostic criteria, such as the Dutch Lipid Clinic Network (DLCN), the Simon Broome criteria [20,21], or Make Early Diagnosis to Prevent Early Deaths (MEDPED) criteria. However, these systems were developed and validated in adults, which limits their applicability in pediatrics and contributes to low diagnosis rates among children [22,23]. Currently, no FH diagnostic scoring system has been fully developed and validated for pediatric populations using real-world data. A study published in 2025 focused on developing FH-PeDS, a novel pediatric diagnostic score. Derived from real-world clinical data, this score aims to enhance both the identification of children likely to have FH for genetic testing and the accuracy of clinical diagnoses in settings where genetic testing is inaccessible or impractical. ML-FH-PeDS offers a flexible diagnostic approach, allowing threshold adjustments to balance sensitivity and specificity based on clinical needs [24].

Different screening strategies are available, with the most common being:

- Cascade screening: A strategy used to identify an index case through genetic testing or clinical criteria, followed by the diagnosis of family members of the index case (e.g., the Dutch model, MEDPED [25,26]
- Universal screening: Involves measuring cholesterol levels in all children and performing genetic testing if cholesterol levels are elevated (e.g., the Slovenian model) [27].

Since FH is significantly underdiagnosed in most countries, leading European experts on FH (European Atherosclerosis Society-EAS) recommend various screening strategies [22,28,29]. The two main strategies are selective (i.e., cascade screening) and population-based (i.e., universal screening). Cascade screening refers to the process where the identification of one patient through screening requires the screening of all family members for the same condition. After diagnosing FH in the proband (index case), or the first family member with a confirmed genetic mutation, it is essential to screen other family members for FH [17]. In population-based (universal) screening, the total cholesterol (TC) concentration is measured in children of a certain age [7]. This approach is currently used in Slovenia, where the screening includes children at the age of 5 or just before starting school.

The most well-known cascade screening program for FH is in the Netherlands, where more than 23,000 patients with FH have been identified through screening. However, the downside of the program is that it fails to reach 30% of the expected FH patient population, as for optimal population coverage, the so-called index case must be identified [30]. In Slovenia, the national cholesterol screening program was gradually implemented, starting in 1995 with the mandatory measurement of total cholesterol (TC) at the age of five, as outlined in the official health leaflet of the Republic of Slovenia. In 2011, routine genetic diagnostics for familial hypercholesterolemia (FH) were introduced at the genetics laboratory of the University Children’s Hospital (UCH) Ljubljana, enabling more accurate identification of FH cases and supporting the further development of the universal FH screening program. In 2017, a brochure with the updated screening algorithm was issued and distributed to primary care pediatricians. In 2019, cascade screening for parents and siblings was introduced (3rd step of screening). The universal screening program is carried out for 5-year-old children during their preventive check-ups. It is estimated that the program reaches 91% of children out of the 96% who undergo the preventive check-ups for 5-year-olds annually [22,27,31].

## 2. Experimental Design

### 2.1. Overview

In Slovenia, a universal screening program for FH is available. Measurement of serum TC levels in preschoolers on primary level is the first step of universal screening. According to national guidelines, children with either serum TC > 6 mmol/L (232.0 mg/dL) or >5 mmol/L (193.4 mg/dL) and a positive family history (defined as known hypercholesterolemia and/or CVD before the age of 60 in a first- or second degree relative) are referred to Pediatric Lipid Clinic at UCH-UMC Ljubljana. At the Pediatric Lipid Clinic, participants’ weight, body mass index, and serum lipid profile (TC, LDL, HDL, and TG) were assessed, and whole-blood samples were collected for targeted next-generation sequencing (NGS) with parental or guardian consent. A custom NGS gene panel of known causative genes for FH (*LDLR*, *APOB*, and *PCSK9*) was developed, enabling comprehensive detection of the majority of pathogenic FH variants. The third step consists of reverse cascade screening for parents and siblings. When a child tests positive for a genetic condition, their parents and siblings are subsequently invited for genetic testing.

This study initially collects comprehensive data from all participating children, regardless of their total cholesterol (TC) levels. Subsequently, children found to have elevated TC at the primary care level will be followed up and referred for further evaluation at the tertiary care Pediatric Lipid Clinic. The process includes detailed lipid profiling, targeted next-generation sequencing for FH-related genes, and reverse cascade screening of parents and siblings. The aim is to evaluate the national screening program, its efficiency, and the outcomes of genetic testing and family screening.

### 2.2. Inclusion Criteria

- Children between the ages of 4.5 and 6 who respond to an invitation to a preventive check-up scheduled at the age of 5 or before entering school in 2023;
- Signed informed consent by parents or legal guardians for genetic testing and cascade screening of parents and sibling;
- In cases where the child has a confirmed positive genetic result, signed informed consent is obtained from the parents prior to performing genetic testing on them.

### 2.3. Exclusion Criteria

- Refusal to do blood sampling on primary level;
- Refusal of genetic testing on tertiary level.

### 2.4. Recruitment

In our study, we perform an analysis of all three steps, focusing on 2023. Pediatricians and school medicine specialists from community health care centers and private outpatient practices were invited to participate. We promoted our study at annual national pediatric conferences and through the primary care pediatric mailing list.

### 2.5. Patient and Public Involvement

Participants were not directly involved in the study design. Since the national screening program was already ongoing, we just collected already obtained data.

## 3. Materials and Equipment

The primary outcome of this study is to evaluate the national pediatric lipid screening program and assess its overall efficiency. Specifically, we aim to investigate and determine participation rates within the program.

Participation rates will be calculated in relation to the overall attendance rates at preventive health examinations for children in Slovenia, as these data are publicly available through the National Institute of Public Health database. In addition, during data collection, we will identify and account for missing data among children who attended the preventive examination but did not undergo blood sampling. These missing data will be categorized into two subgroups: (1) children who were referred for laboratory testing but did not complete it (typically due to parental or guardian decision), and (2) children who were not referred for laboratory testing by their pediatrician. This approach will allow us to distinguish between procedural and behavioral factors contributing to incomplete screening participation.

The secondary outcomes are as follows:

- Percentage of children with elevated TC;
- Percentage of children referred to tertiary clinic;
- Percentage of children with positive genetic testing;
- Percentage of families screened, and additional family member who confirm FH.

### 3.1. Measurement of Serum Cholesterol

Serum cholesterol is measured from a capillary or venous blood sample. As mentioned above, the study involves both public institutions and private outpatient practices, meaning that cholesterol measurement devices may vary between institutions. Participating institutions provided information about the type of cholesterol measurement device used when submitting data.

### 3.2. Algorithm for Implementing the Cholesterol Screening Program

**Figure 1:**
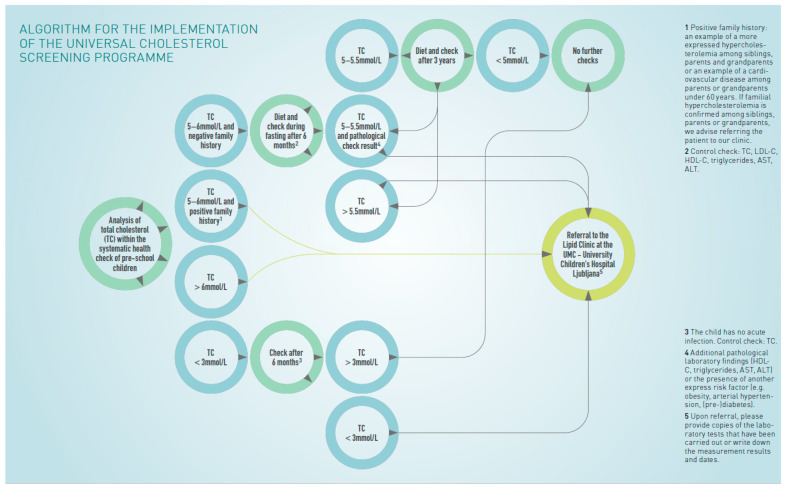
Algorithm for the implementation of the universal cholesterol screening program.

The current algorithm (Figure 1) applies to children undergoing the preventive check-up. If serum cholesterol is above 6 mmol/L (232.0 mg/dL) or between 5 and 6 mmol/L (193.4–232.0 mg/dL) and the family history is positive, the child is referred to the Pediatric Lipid Clinic UCH-UMC. For values between 5 and 6 mmol/L (193.4–232.0 mg/dL) and a negative family history, a follow-up will be scheduled after 6 months. Repeated check-ups on primary care should include an extended lipid profile (TC, LDL, HDL, TG) and liver function tests (aspartate transaminase (AST) and alanine transaminase (ALT)). If again TC levels are between 5 and 5.5 mmol/L (193.4–212.7 mg/dL) and other pathological findings are found, or levels exceed 5.5 mmol/L (212.7 mg/dL), the child should be referred to the Pediatric Lipid Clinic UCH-UMC. For values between 5 and 5.5 mmol/L (193.4–212.7 mg/dL), an informative suggestion for dietary instructions will be provided. The next follow-up is planned after 3 years. If levels are above 5.5 mmol/L (212.7 mg/dL), the child will be referred to the Pediatric Lipid Clinic UCH-UMC; otherwise, no further monitoring is necessary. The algorithm also includes the management of children with low TC levels. Children with TC levels below 3 mmol/L (116.0 mg/dL), a follow-up scheduled after 6 months. If the value remains below 3 mmol/L (116.0 mg/dL), the child will also be referred to the Pediatric Lipid Clinic UCH-UMC. If levels are not persistently low, no further follow-up will be needed. The algorithm also summarizes general dietary guidelines for elevated TC.

### 3.3. Statistical Methods

#### 3.3.1. Sample Size Calculations

Our goal is to collect data from 5000 children at the primary care level; the subsequent step will include follow-up of all referred children at the tertiary clinic. The study population consists of children born in 2017 (screened before entering school) and in 2018 (screened at the 5 year preventive check-up). In Slovenia, there were 20,241 live births in 2017 and 19,585 in 2018. Thus, a sample of 5000 children represents approximately one-quarter of a single birth cohort. Based on the expected prevalence of FH (1:313–1:400), we anticipate confirming FH in approximately 12–15 children.

#### 3.3.2. Statistical Analysis

All statistical analyses will be performed using IBM SPSS Statistics 28.0.0.0. Descriptive statistics, including medians, interquartile ranges, and proportions with 95% confidence intervals, will be reported for demographic variables, and the prevalence of FH will be calculated with 95% confidence intervals, stratified by sex.

The central thesis of this study—that universal screening enables the detection of FH in the majority of children within a defined age group—will be tested. It is anticipated that FH will be confirmed in approximately 15 children out of 5000 screened, that screening coverage will exceed 90%, and that no significant sex differences in FH prevalence will be observed. Concordance between FH status in children and their parents will be assessed, and the association between a positive family history of premature cardiovascular disease and confirmed FH in the child will be evaluated. Appropriate statistical tests, including the chi-square test, logistic regression, one-sample proportion test, and McNemar’s test, will be applied as appropriate, with *p*-values < 0.05 considered statistically significant.

## 4. Detailed Procedure

**Figure 2:**
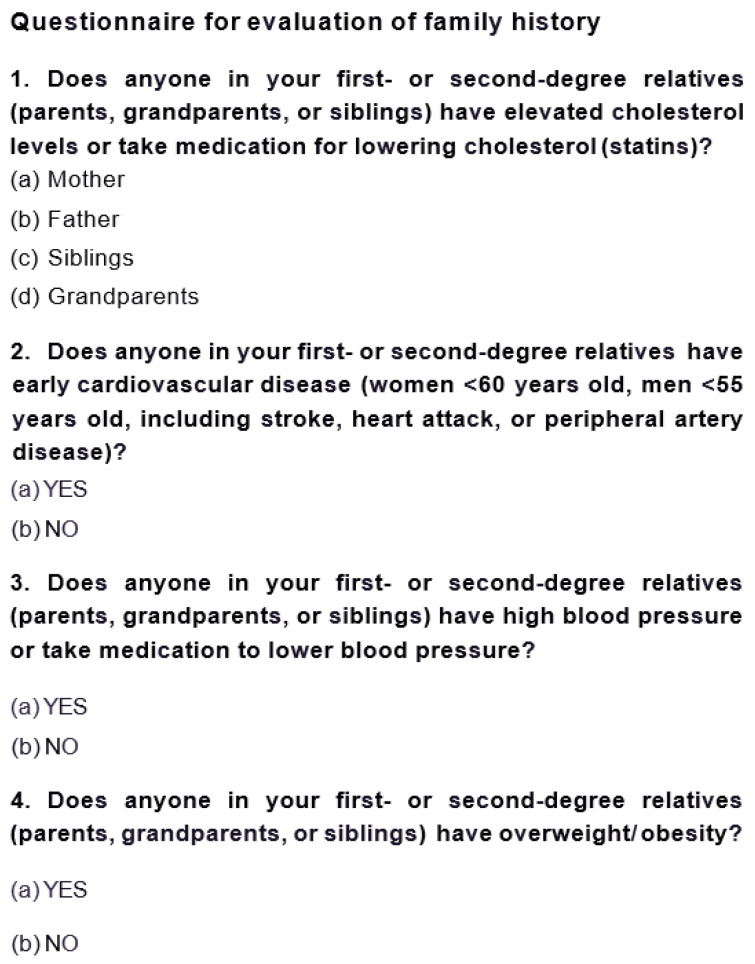
Questionnaire for the evaluation of family history of premature cardiovascular complications. This questionnaire was sent to primary care pediatricians involved in the prospective phase of the study.

The study takes place in Slovenia. Before the start of data collection, written consent for institutional participation was obtained from all primary care facilities involved in the study. Primary care pediatricians have already been supported in their work by an existing algorithm for implementation of a universal cholesterol screening program (Figure 1). Additionally, we provided a questionnaire to easily determine a positive family history of familial hypercholesterolemia in parents and grandparents, as well as the presence of other cardiovascular risks (Figure 2).

During the preventive check-up of preschool children (4.5–6 years), parents are informed about familial hypercholesterolemia and the significance of laboratory blood tests for TC levels with the aim of enhancing public awareness of FH.

The preventive check-up includes the collection of the following data: date of birth, date of examination, gender, age, height, weight, waist circumference (WC), TC levels, family history (positive family history: significant hypercholesterolemia in siblings, parents, or grandparents, or a history of cardiovascular disease in parents or grandparents before the age of 60).

In the prospective phase of the study, participating primary care institutions completed the questionnaire themselves (Figure 2) and entered the required data listed above. In the retrospective phase, the research team visited additional primary care centers to supplement the overall dataset. It should be emphasized that the electronic health record system and electronic data entry infrastructure are still underdeveloped in Slovenia. Consequently, most data were manually transcribed by the research team from physical (paper-based) medical records. Missing or incomplete information—such as laboratory referrals or TC values—was verified and supplemented through review of the available electronic records.

In accordance with national guidelines for FH, children presenting with either a serum total cholesterol (TC) level > 6 mmol/L, or >5 mmol/L in the presence of a positive family history, are referred to the Pediatric Lipid Clinic at UCH-UMC.

**Figure 3:**
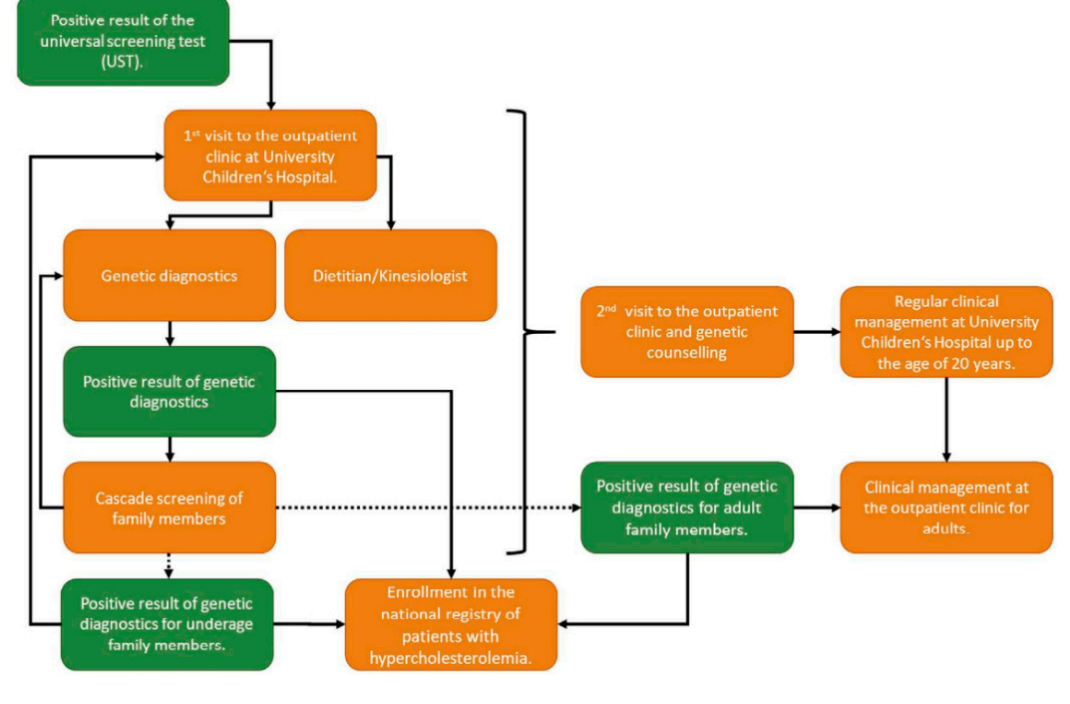
Algorithm of the genetic FH program (second step) and subsequent clinical management of referred children to tertiary care level (UHC Ljubljana).

During the initial clinical evaluation in the Pediatric Lipid Clinic at UCH-UMC, anthropometric measurements (weight, height, BMI calculation, waist circumference) are obtained, and blood pressure as well as heart rate are recorded. In addition, a blood sample is collected to assess an extended lipid profile (TC, LDL, HDL, TG) along with whole-blood samples for targeted NGS (Figure 3) [32,33].

If genetic analysis confirms a pathogenic variant associated with familial hypercholesterolemia, parents and siblings are invited to undergo genetic testing. In cases where familial hypercholesterolemia is diagnosed in parents or siblings older than 20 years, they are referred to a lipidologist at a tertiary clinic. For siblings under 20 years old, management is continued at the Pediatric Lipid Clinic at UCH-UMC. If genetic analysis does not identify a causal variant, the condition is most likely attributable to polygenic hypercholesterolemia or hypercholesterolemia of unknown origin, and treatment is guided by the clinical presentation. So far, we have results from a pilot child–parent cascade screening program; 138 parents from 123 families underwent genetic testing. Among 123 parents of index pediatric cases, (likely) pathogenic variants were detected in 95 (77.2%). In families where the initially tested parent was negative (*n* = 15), testing the other parent identified variants in 14 (93.3%). Overall, cascade screening was successful in 109/110 families (99.1%), as only one family had no variant detected in either parent [34].

## 5. Expected Results

In our study, the central thesis is that the existing universal screening program for FH enables the detection of FH in most children of a certain age group.

The hypotheses that support this thesis are as follows:

- We expect that FH will be detected in 15 children (out of 5000 included in the study) between the ages of 4.5 and 7 years.
- We expect that more than 90% of all children included in the preventive health check will undergo screening for FH.
- We expect an equal distribution of FH occurrence between both sexes.
- We expect that for each child with confirmed FH, the condition will also be confirmed in one of the parents.
- We expect that a positive family history of premature CVD will positively correlate with confirmed FH in the child.

Participation rates will be calculated as the proportion of children who took part in the screening program out of the total number of eligible children invited to the preventive check-up. For this analysis, we will include:

- Children who were not referred for TC testing by their pediatrician or school medicine specialist.
- Children who were referred for TC testing but did not undergo the test for various reasons.
- Children who were referred to the tertiary clinic but did not participate (due to refusal of additional testing or other reasons).
- Children who did not attend the preventive check-up.

This comprehensive approach will allow us to clearly determine the true participation rate and understand barriers to participation at each stage of the screening process.

To date, no study has simultaneously evaluated all three steps of the population-based screening program (screening of total cholesterol at the primary care level; genetic testing of children with elevated cholesterol levels; and cascade genetic testing of parents). This investigation will provide the most robust estimate to date of the prevalence of FH in Slovenia. Furthermore, it will allow an assessment of the proportion of the pediatric population reached by the screening program and determine whether the existence of a national screening initiative in Slovenia leads to a higher diagnostic yield of FH compared to other countries.

The findings will enable an evaluation of the effectiveness of cholesterol screening in Slovenian preschool children, thereby confirming key hypotheses, and will provide evidence to guide potential refinements of the screening program as well as updates to the national guidelines for its implementation.

## Data Availability

No new data were created or analyzed in this study.

## Abbreviations

UCH-UMC	University Children’s Hospital, University Medical Center
FH	Familial hypercholesterolemia
CVD	Cardiovascular disease
ASVD	Atherosclerotic cardiovascular disease
DALY	Disability-adjusted life year
TC	Total cholesterol
LDL	Low-density lipoprotein
HDL	High density lipoprotein
TG	Triglyceride
LDLR	Low-density lipoprotein receptor
HoFH	Homozygous familial hypercholesterolemia
APOB	Apolipoprotein B
PCSK9	Proprotein convertase subtilisin/kexin type 9
DLCN	Dutch Lipid Clinic Network
MEDPED	Make Early Diagnosis to Prevent Early Deaths
FH-PeDS	Familial Hypercholesterolemia Pediatric Diagnostic Score
ML-FH-PeDS	Machine learning model of Familial Hypercholesterolemia Pediatric Diagnostic Score
EAS	European Atherosclerosis Society
NGS	Next generation sequencing
WC	Waist circumference
AST	Aspartate transaminase
ALT	Alanine transaminase
BMI	Body Mass Index
